# Emergency remote teaching technology and pedagogy at covid outbreak: different perspectives of students, parents, and teachers in Hong kong

**DOI:** 10.1007/s10639-022-11526-2

**Published:** 2022-12-28

**Authors:** Kit-Tai Hau, Wen Jie Wu, Wing Tung Chung, Sze Ching Chan, Ming Ho Ng

**Affiliations:** 1 Department of Educational Psychology, The Chinese University of Hong Kong, Hong Kong SAR, PR China; 2 School of Psychological Science, The University of Newcastle, Newcastle, Australia

**Keywords:** COVID-19, Digital learning, Information technology, Hong Kong, Emergency remote teaching

## Abstract

With the COVID-19 outbreak, emergency remote teaching – an unprepared distant mode of education became the only possible alternative for schools. The present large-scale survey with 3,672 Grade 3 and 9 students, their parents, and 863 teachers/principals was conducted in the metropolitan city of Hong Kong after half a year of school lockdown. Results showed teachers, principals, and parents were worried about students’ inability to concentrate and learn without teachers’ explanations. In contrast, students, particularly younger ones, were less affected. They perceived their academic achievement was not worsened and they were more lively. Generally, lack of computers and stable internet was not seen as problems. Notably, socially disadvantaged students were not different in their perceived challenges, affects, life satisfaction, or perceived academic achievement. For cities with adequate provision of computers and internet facilities, the pandemic probably forced a positive and giant leap in using advanced technologies and pedagogies.

## Introduction

Not only medical professionals but people in the whole world were affected by COVID-19. Understandably, student academic learning was disrupted during the closure of the school premises. A simulated study by the World Bank over 157 countries suggested that 3 to 5 months of school closure would result in an optimistic to pessimistic loss of 0.3 to 0.9 years of schooling (Azevedo et al., 2020). Despite the global panic, some people might see this as an excellent opportunity to promote digital learning. The pandemic became a giant unplanned no-control-group global experiment to examine how well digital remote teaching could replace traditional face-to-face classroom instruction.

Some academic studies on COVID’s immediate effects on learning, usually small-scale ones, have already been collated into special journal issues on the impact during the first few months of the pandemic (Abdel-Hameed et al., 2021; Reuge et al., 2021; Starkey et al., 2021). In contrast, we used a large representative sample of students, teachers, parents, and principals in Hong Kong in this study to examine the possible impacts of COVID on different aspects of students’ learning.

## Literature Review

### Education and Pedagogies during the pandemic

During the unprecedented sudden pandemic disruption, United Nations (2020) estimated that 94% of students worldwide were affected. With lockdown and social distancing, remote teaching became the only option available for schools. This emergency remote teaching distinguished itself as (a) a temporary and sudden shift without alternatives, (b) a fully remote mode of teaching in place of face-to-face or blended instruction, (c) an unprepared mode without any pretence to become a robust, long-lasting system, and (d) quick-to-set-up temporary access to learning (Bozkurt et al., 2020; Hodges et al., 2020).

Surveys reaffirmed the wide adoption of such emergency remote teaching around the world. In 2020, the Organisation for Economic Co-operation and Development (OECD, 2020) consortium Education Policy Outlook (EPO) examined over 40 major education systems on how they were affected and responded to the pandemic between July and September 2020, about the time of the present study. Understandably, schools were chaotic in the earlier half of 2020. By September 2020, most governments had a central official policy of remote or hybrid instruction delivery (e.g., prerecorded material was more prevalent in some cities).

Obviously, we expect emergency remote teaching to work better in systems well provided with ICT before the pandemic. Among academically high-performing economies, Hong Kong was average to slightly low in ICT availability at home but slightly above average in school (OECD, 2019). On a scale of 0 to 10 for ICT availability at home, Hong Kong (7.71) was lower than Finland (8.31), OECD average (8.17), and Estonia (8.13) but similar to Singapore (7.81), Macao (7.72) and Taipei (7.52). In terms of ICT availability at school, Hong Kong (7.16) was similar to Finland (7.19) but higher than Singapore (6.78), Estonia (6.47), Macao (6.37), OECD average (6.28), and Taipei (5.96). Access to the internet at home in Hong Kong has been consistently high (96.9–97.6% from 2006 to 2018). On the use of ICT in teaching, among the high-achieving economies, Hong Kong was slightly more frequent in using ICT outside of school but on average in using ICT at school (OECD, 2019).

### Impacts on learning during the pandemic

Without face-to-face instruction, students encountered different sorts of problems. For young students and kindergarteners, their parents worried about the lack of learning atmosphere and social interaction with online learning (Lau & Lee, 2020). Even the more technology-competent university students were quite anxious (Unger & Meiran, 2020). They felt the quality of teaching was compromised (Ionescu et al., 2020), their learning was disruptive (Mollenkopf et al., 2020), and there was a lack of student-teacher interaction and socialization with peers (Ionescu et al., 2020).

Despite the general worry about students’ learning during the pandemic, people could see some positive aspects of emergency remote teaching (Alabdulaziz, 2021). These included ‘ease of online courses, flexibility in the work schedule, adaptability to broad learning styles, a variety of tools available at hand, ease in monitoring, and documenting teaching activities’ (Ionescu et al., 2020, p.12). Even during the COVID outbreak, when schools were chaotic, parents in the USA were still quite satisfied with the school arrangement (Cullinane & Montacute, 2020). Parents with children learning at home (61% satisfied) were similarly satisfied as those learning in schools (65%).

Despite the initially greater concerns, students and parents became more positive about the pedagogy change once they were adapted to the new learning mode. Students gradually felt they had the necessary technology for ‘distant’ learning (Mollenkopf et al., 2020), found it easy to access the resources needed to complete their assignments (Azis & Fatimah, 2020; Blankstein et al., 2020), had greater confidence in online learning, and planned their learning more systematically (Gonzalez et al., 2020).

Students might not find their learning situation under the pandemic as bad as perceived by the teachers, parents, or the public. Thus, for example, even in Asia, a survey of Malaysian university students showed that many students (85%) rated their experience as acceptable (OK to Very good) in March 2020, when the pandemic had just started and increased to almost all students (91%) half a year later. This reflected students’ willingness to accommodate their learning when alternatives were unavailable.

### Impacts on self-management and Social Development

Students would be more ready for emergency remote teaching when they could self-monitor their learning without continuous teachers’ supervision. Students’ loss of focus and motivation was common in the absence of teachers’ constant supervision (Driessen et al., 2020).

During the pandemic, students were likely more stressed and had more mental health problems (Blankstein et al., 2020). In Hong Kong SAR, China, one-third of secondary school students were estimated to be mentally distressed (Li & Leung, 2020). Not only students but parents also were stressed. Around the world, parents were overburdened with financial difficulties (Blankstein et al., 2020), irregular hours in working from home, and “home-schooling” for their children. Parents became teachers to supervise their children’s study at home (Bozkurt et al., 2020). This often resulted in psychological pressure and tension for both parents and children.

On the positive side, some students believed the pandemic could help them strengthen their self-directed learning competence and time management, enhancing their preparation for future e-working environments (Al-Naimi et al., 2021).

### Digital divide -- Widening of Social Division

When students were forced to take an emergency distance mode of education, the widening of social disparities became an imminent concern (Reuge et al., 2021 and related articles in that special issue). Learning at home provided flexibility, but it worked only when students had the necessary space, equipment, and environment at home. Without teachers’ supervision, students might be unable to balance multiple responsibilities and tasks, such as homework, preparation, revision, and other activities (Mollenkopf et al., 2020). For students living in relatively congested metropolitan areas, finding a quiet space to study at home was difficult. Even university students who could travel independently had difficulty finding a quiet place to study during the pandemic (Blankstein et al., 2020). Furthermore, materialistic issues were more severe with disadvantaged students, affecting their academic performance.

Given the additional problems of the pandemic on disadvantaged students, the pandemic would likely increase the social divide. Surveys estimated that one-third of children globally (particularly in developing countries) or disadvantaged Latino students in the USA would have inadequate computer or internet facilities for educational use during the pandemic (Kim & Padilla, 2020). It was not surprising, therefore, to see low socio-economic and female students in Australia (Dodd et al., 2021), disadvantaged groups in the USA (women, non-Hispanic Asian, fair/poor health, below-average family income, families losing income) (Aucejo et al., 2020), disadvantaged students in China [living alone (vs. living with families), living in rural (vs. urban), low family income, with a relative/acquaintance affected with COVID-19] (Cao et al., 2020), and students from working-class (vs. middle-class ones)(Cullinane & Montacute, 2020) were affected more by the pandemic on their learning resources, academic performance, and psychological well-being. Given primary and secondary school students’ greater reliance on the schools and being less IT competent, their problems and social divide were likely more severe than the tertiary ones (Bozkurt et al., 2020).

In sum, it was postulated in this study that high socioeconomic status could be an essential facilitating factor in students’ learning. Online access, social support, and the change in teaching pedagogies during the pandemic would likely increase the digital divide, particularly for younger children.

### Differential Cultural Impacts

Asian students have been shown to perform outstandingly in earlier studies (Hau & Ho, 2010) and more recent international surveys such as PISA (OECD, 2019). Probably it is because education is highly valued in the Confucian tradition. Students study hard to uphold a cluster of valued attitudes, including striving to enhance the family’s status, emphasis on effort (vs. ability), belief in persistence until success, and diligence as an obligation to parents and the family (Hau & Ho, 2010; Li, 2005).

We also predicted that parental support might be stronger in Asian or Chinese cultures (Hau & Ho, 2010). Cheung and Pomerantz (2011) compared American and Chinese children (mean age 12.74 years) on their parents’ involvement. Results suggested American parents tended to provide greater autonomy support and had less control than their Chinese counterparts. Thus, given the stronger Asian familial support, the impact of the pandemic on Asian students could be much smaller than their western counterparts. Furthermore, parents played the role of surrogate teachers, particularly for younger children whose subject contents were relatively easy for the parents. Thus, negative impacts on younger children would likely be smaller.

### Education in Hong Kong during the pandemic

Educators and researchers were similarly worried about COVID-19 impacts on Hong Kong students (Lee, 2020). At the beginning of the pandemic (February, 2020), Lau and Lee (2020) surveyed a sizeable convenient sample (N = 6,702) of parents of kindergarten and primary school students. They suggested that most children had difficulties and could not learn independently at home. However, it was uncertain whether it was the parents’ over-concern or a true reflection of the chaotic learning.

About three months after the school premise closure (May 7–12, 2020), Mok et al. (2021) surveyed a sizeable convenient sample (N = 1,227) of tertiary students in Hong Kong. Results showed that despite students’ preference for face-to-face teaching, they were not too negative in their satisfaction with online learning. Students with poor information technology proficiency and lower family income were more dissatisfied. This was congruent with another survey of 425 tertiary students in Hong Kong (collected in April, 2020) (Ho et al., 2021). Over 90% of the students reported having stable WiFi connections and high self-efficacy in digital competence.

Immediately after the outbreak of COVID-19, Ng et al.‘s (2020) naturalistic inquiry into suggested that the centralized IT support of these institutes was adequate and could help teachers start their online learning and video-recording lessons. The new teaching pedagogies adopted in the pandemic could be desirable even when regular teaching could be resumed. Ng et al. (2020) and Ng and Chu (2021) examined artificial intelligence teaching during COVID-19 using a social networking site and synchronous online sessions. They found that it was as effective as the traditional face-to-face classes.

Here, we provide some contextual factors on the impact of COVID on Hong Kong at the time of data collection. When the World Health Organization (WHO) declared the outbreak of the novel coronavirus infection on January 30, 2020, Hong Kong students were close to having their long Chinese New Year holiday (lunar new year on February 12, 2020). After the holidays, Hong Kong school buildings continued to be closed, with lessons delivered through web-based distant mode. Around four months later, students gradually returned to school for half-day on some days of the week, starting late May to early June 2020. The main batch of the present survey was conducted in mid- or late June, soon after students had returned to the schools. Medically, COVID’s impact on Hong Kong was minimal. By June 1, 2020, with a population of over 7.5 million people, there were only around 1000 identified COVID cases, with four deaths.

### The Present Study

In contrast to the above studies, the present study took place slightly later, about half a year (June - July 2020) after the pandemic outbreak, when students and teachers became more familiar with emergency remote teaching. A much larger representative sample of students, their parents, and teachers/principals in Hong Kong participated in the present study. We compared their perception of academic progress, challenges, and affect. Importantly, we also examined whether learning problems were aggravated among disadvantaged students or low academic achievers. In Hong Kong, the information-community technology (ICT) availability was average compared to other high-academic-performing economies (OECD, 2019). We were interested to know how well such a learning system survived during the pandemic.

## Methods

### Participants

In this cross-sectional study, a total of 2,019 Grade (G.) 3 students with their parents, 537 teachers, and the respective principals of G.3 students from 33 schools (around 4% of the Hong Kong population); as well as 1,653 G.9 students (with their parents), 326 teachers and respective principals of G.9 students from 24 schools (around 3.5% of the Hong Kong population) participated and completed a questionnaire survey as part of a government-commissioned project.

About half of the students (53% and 47% of G.3, G.9) also completed achievement tests on Chinese, English, and mathematics, the three most important academic subjects in the school curriculum. A disproportionate stratified method was adopted with school size (small, medium, large) and school finance type (aided, government, private, private/direct subsidy scheme) as stratification criteria. Sampling weights were applied so that the current results were representative of the Hong Kong total student population, with the exclusion of around 6% of international school students.

For some questionnaire items, we compared the responses of another representative batch of students collected one year ago (before the pandemic, June 2019). There were 10,064 G.3 students with their parents from 398 schools and 4,445 G.9 students with their parents from 55 schools in this comparison group. Again appropriate sampling weights were applied so that the results represented the Hong Kong population (other than international schools).

### Measures

We also referred to the OECD PISA Global Crises Questionnaire Module (Bertling et al., 2020) in constructing the instruments for the present study. Thus, for example, we asked students to compare their learning when the school building was closed (learned less, the same, or more). We also asked for a list of other common problems/challenges, including access to digital devices, internet access, a quiet place to study, motivating myself, and finding someone to help.

Though students, parents, teachers, and principals answered different sets of questionnaires, some items were purposely designed in parallel forms. For example, parents, teachers, and principals were asked parallel sets of items on their perception of students’ challenges, affect, and expected changes in academic achievement.

#### Students’ challenges during the pandemic

Four groups of participants (students, parents, teachers, and principals) were asked how often students encountered nine types of challenges when they studied at home during school closure (“Were the following factors causing problems when (you/your students/your child) studied at home during class suspension?”). These challenges included (i) having no computer to work with, (ii) lack of a stable/fast enough internet connection, (iii) lack of a quiet place to study, (iv) difficulty in understanding the content without the teacher’s help on the spot, (v) inability to concentrate on study, (vi) worry about a virus infection, (vii) worry about the pandemic impacts on family income, (viii) relation problems with parents, (ix) no person at home to help. These nine items items were on 5-point scales (1 ‘no, never a serious problem’ to 5 ‘yes, the problem occurs many times a day’).

#### Teachers’ challenges during the pandemic

Teachers were asked how often they encountered five types of challenges when planning learning activities for students during school closure (“Were the following factors causing problems when you planned learning activities for your students during class suspension?”). The five challenges included ‘lack of support from the principal,’ ‘lack of support from middle management,’ ‘lack of school-based policy support measures,’ ‘lack of computer skills,’ and ‘technical staff lacking skills to support.’ These five items were on 5-point scales (1 ‘no, never a problem’ to 5 ‘Yes, a problem with almost all situations’).

#### Students’ affect before and during the pandemic

Students, parents, teachers, and principals were asked to rate how often students felt ‘happy,’ ‘lively,’ ‘afraid,’ and ‘stressed’ (i) before and (ii) during the pandemic [“How often did (you/your child/your students) feel as described below before and during class suspension?”]. These four items imitated or were adopted from the Programme for International Student Assessment (PISA; OECD, 2019) and were on 4-point scales from 1 ‘never’ to 4 ‘always.’

#### Expected changes in academic achievement

Students, parents, teachers, and principals were asked to rate their expected changes in students’ Chinese language, English language, and mathematics academic achievement upon returning to school [“Upon returning to school, do you think (your/your child’s/your students’) study performance is worse, similar, or better than that you could have been in usual lessons?”]. These three items were on 9-point scales (1 ‘very much worse’ to 9 ‘very much better’).

#### Students’ life satisfaction

Students were asked how satisfied they were in different aspects of life, including health, knowledge or skills learned from school, friendship, time usage, relationship with parents, relationship with teachers, and things they had (“During class suspension, how satisfied were you with each of the following?”). These seven items were adopted from PISA (OECD, 2019) and were on 4-point scales (1 ‘not at all satisfied’ to 4 ‘totally satisfied’).

#### Socioeconomic status (SES)

Family SES was measured by aggregating five standardized items/variables in the parent questionnaire: father’s and mother’s educational level, father’s and mother’s occupational status, and monthly family income (Cronbach’s α 0.87 and 0.82 for G.3 and G.9, respectively). The standardization was conducted within each educational level and academic year separately.

#### Academic achievement

Three high quality academic achievement tests in Chinese, English, and mathematics, respectively, based on the local school curriculum were constructed by the respective subject and assessment expert committees. Students’ scores in each academic subject were standardized within each educational level. The mean of three standardized achievement tests was used as an indicator of students’ overall academic achievement.

### Data analyses

In the present study, we examined during school closure (i) whether students, parents, teachers, and principals had a similar view on students’ challenges and affect, (ii) whether students’ challenges and affect were associated with their socioeconomic status and academic achievement, (iii) the prevalence of challenges encountered by teachers, (iv) whether students, parents, teachers and principals had a similar view on changes in students’ academic achievement, (v) whether students’ perceived changes in academic achievement were associated with their socioeconomic status and academic achievement, (vi) whether students’ life satisfaction was affected. ANOVA or ANCOVA was conducted to compare responses from students, parents, teachers, and principals for the parallel sets of questionnaire items. As the sample sizes of different groups of participants were drastically different, we relied more on posthoc pairwise tests to draw conclusions. To reduce Type I errors in multiple comparisons, Benjamini–Hochberg correction (Benjamini & Hochberg, 1995) was applied with the false discovery rate set at 0.05. As students’ data were nested within schools, multilevel regression analyses were conducted using R lme4 in examining and controlling for the effects of SES and achievement on various measures (e.g., challenges and affects).

## Result

### Students’ challenges during the pandemic

Descriptive statistics of students’ challenges as perceived by four groups of participants (students, parents, teachers, and principals) were summarized (Table 1). One-way ANOVA was conducted to examine whether these four groups of participants differed in their perception of students’ challenges during the pandemic (Table 2).

**Table 1:** Descriptive Statistics of Perceived Students’ Challenges, Affect Before and During the Pandemic and Expected Changes in Students’ Academic Achievement by Students, Parents, Teachers, and Principals

	Grade 3								Grade 9							
	Students (*n* = 2,019)		Parents (*n* = 2,019)		Teachers (*n* = 537)		Principals (*n* = 40)		Students (*n* = 1,653)		Parents (*n* = 1,653)		Teachers (*n* = 326)		Principals (*n* = 27)	
	*M*	*SD*	*M*	*SD*	*M*	*SD*	*M*	*SD*	*M*	*SD*	*M*	*SD*	*M*	*SD*	*M*	*SD*
**Challenges to Students During the Pandemic**																
Lack: computers	1.25	0.79	1.26	0.74	1.93	1.20	1.50	0.92	1.21	0.70	1.23	0.77	1.89	1.18	1.64	0.89
Lack: stable internet	1.66	1.07	1.53	0.96	2.49	1.26	2.05	1.22	1.67	1.12	1.63	1.08	2.49	1.22	2.35	1.22
Lack: quiet place to study	1.67	1.17	1.82	1.24	2.40	1.21	2.07	1.27	1.65	1.13	1.56	1.04	2.53	1.26	2.19	1.01
Lack: teachers’ explanation	1.90	1.20	2.62	1.34	2.97	1.20	2.69	1.27	2.11	1.20	2.32	1.25	3.07	1.20	2.95	1.19
Concentration on study	1.96	1.27	2.90	1.40	3.36	1.21	2.90	1.33	2.21	1.32	2.39	1.37	3.72	1.15	3.50	1.29
Viral infection	2.11	1.42	1.96	1.17	1.76	1.08	1.62	0.96	1.63	1.13	1.79	1.19	1.65	1.00	1.48	0.72
Family income	2.05	1.40	1.94	1.28	1.67	0.93	1.68	0.91	1.66	1.10	1.78	1.20	1.68	0.89	1.82	1.00
Relation with parents	1.48	0.98	1.69	1.09	1.97	1.02	1.78	1.03	1.32	0.80	1.43	0.90	1.96	0.99	2.18	1.13
No person at home to help	1.40	0.94	1.75	1.18	2.69	1.20	2.63	1.08	1.33	0.85	1.54	1.07	2.22	1.15	2.31	1.10
**Affect Before the Pandemic**																
Happy	3.29	0.82	3.49	0.61	3.48	0.53	3.67	0.48	3.14	0.72	3.27	0.71	3.30	0.51	3.41	0.50
Lively	2.10	0.98	3.52	0.61	3.52	0.54	3.67	0.47	2.61	0.83	3.20	0.73	3.27	0.55	3.38	0.50
Worry	3.19	0.87	2.23	0.81	2.48	0.64	2.66	0.57	3.25	0.71	2.41	0.80	2.51	0.62	2.57	0.50
Stress	2.43	1.06	2.43	0.82	2.71	0.64	2.67	0.53	2.73	0.91	2.59	0.83	2.65	0.65	2.77	0.57
**Affect During the Pandemic**																
Happy	3.30	0.84	3.41	0.63	3.26	0.59	3.20	0.66	3.03	0.78	3.25	0.69	3.06	0.60	2.95	0.57
Lively	2.25	1.06	3.29	0.70	2.92	0.69	2.97	0.66	2.81	0.85	2.98	0.77	2.67	0.70	2.78	0.63
Worry	3.17	0.88	2.39	0.87	2.66	0.73	2.91	0.64	3.03	0.81	2.56	0.85	2.73	0.74	3.00	0.57
Stress	2.36	1.10	2.33	0.90	2.55	0.72	2.94	0.65	2.73	0.94	2.60	0.88	2.77	0.77	3.01	0.62
**Expected Changes in Academic Achievement**																
Chinese language	5.39	2.01	4.42	1.86	3.61	1.34	4.18	1.29	4.82	1.57	4.62	1.61	3.96	1.51	4.40	1.40
English language	5.39	2.07	4.39	1.90	3.27	1.35	3.81	1.48	4.72	1.63	4.58	1.58	3.70	1.54	3.93	1.34
Mathematics	5.87	2.04	4.78	1.88	3.53	1.35	3.99	1.23	4.65	1.86	4.64	1.77	3.80	1.51	4.18	1.60

*Note*.

Challenges to students on a 5-point scale (1 = no, never a serious problem, 5 = yes, a problem many times a day);

Affect before/during the pandemic on a 4-point scale (1 = never, 4 = always);

Expected changes in academic achievement on a 9-point scale [1 = worse than usual lessons in schools (poor), 5 = about the same as usual lessons in schools; 9 = better than usual lessons in schools (good)]

We focused on medium or large effects (η^2^ = 0.06, 0.14 respectively, Cohen, 1988), followed by the Tukey HSD test with Benjamini–Hochberg correction. In general, on students’ issues, teachers, principals, and parents felt “students’ lack of concentration on study” (G.3, η^2^ = 0.14; G.9, 0.09) and “lack of teachers’ face-to-face explanation” (G.3, 0.09) were more serious than students did. On environmental factors, teachers and principals generally felt ‘no person at home can help’ (G.3,0.12; G.9,0.06), ‘lack of computers’ (G.3, 0.07; G.9, 0.06), ‘lack of stable internet’ (G.3, 0.07), and ‘lack of a quiet place to study’ (G.9, 0.06) more serious than students and parents did.

Particularly to students and parents, ‘lack of computers’ and ‘lack of stable internet’ were the least or among the less concerned challenges (Table 2).

**Table 2:** ANOVA of Students’ Challenges During the Pandemic Perceived by Person (Students, Parents, Teachers and Principals)

	Grade 3						Grade 9				
	*SS*	*df*	*MSE*	*F*	η_p_^2^		*SS*	*df*	*MSE*	*F*	η_p_^2^
Lack: computers	220.64	3.00	73.55	107.04	.07		139.71	3.00	46.57	75.93	.06
Lack: stable internet	398.55	3.00	132.85	120.09	.07		222.75	3.00	74.25	60.04	.05
Lack: quiet place to study	229.12	3.00	76.37	52.62	.03		266.85	3.00	88.95	73.69	.06
Lack: Teachers' explanation	767.08	3.00	255.69	160.76	.09		263.02	3.00	87.67	58.40	.05
Concentration on study	1298.69	3.00	432.90	248.57	.14		663.02	3.00	221.01	124.74	.09
Virus infection	64.96	3.00	21.65	13.29	.01		23.04	3.00	7.68	5.88	.00
Family income	63.51	3.00	21.17	12.62	.01		12.67	3.00	4.22	3.30	.00
Relation with parents	113.13	3.00	37.71	35.45	.02		127.73	3.00	42.58	56.50	.04
No person at home to help	743.89	3.00	247.96	211.67	.12		237.22	3.00	79.07	81.58	.06

*Note*. *SS =* Sum of Squares; *df =* degrees of freedom; *MSE =* Mean Square error; η_p_^2^ *=* Partial eta squared.

### Social and Digital divide

To understand whether the social and digital divide was magnified during the pandemic, we also examined how these challenges were related to students’ SES and academic achievement in multilevel regression models (Table 3). Results showed that apart from family income (G.3: *β* = − 0.08, *p* < .05), disadvantaged students did not perceive any of the challenges to be more severe than other students; *β* ranged from − 0.01 to − 0.06 and 0.00 to − 0.07 for G.3 and G.9 respectively. However, students with low academic achievement perceived some challenges as more severe than students with high academic achievement. G.3 low achievers perceived all challenges, except viral infection, to be more severe than high achievers; *β* ranged from − 0.10 to − 0.18; for viral infection, *β* = − 0.04, *p* = .12. In G.9, low achievers perceived relation problems with parents (*β* = − 0.13) and no person at home to help (*β* = − 0.08) to be more severe than high achievers.

**Table 3:** *Multilevel Regression Predicting Students’ Challenges During the Pandemic with SES and Academic Achievement*

	no computers		no stable internet		no quiet place to study		no teachers explain		cannot concen-trate in study		virus infection		worry family income		relation with parents		no person at home help	
	*β*	*p*	*β*	*p*	*β*	*p*	*β*	*p*	*β*	*p*	*β*	*p*	*β*	*p*	*β*	*p*	*β*	*p*
Grade. 3																		
SES	− 0.01	0.76	− 0.06	0.06	0.01	0.73	− 0.01	0.73	− 0.03	0.27	− 0.01	0.63	− 0.08*	0.01^1^	− 0.05	0.10	− 0.02	0.55
achievement	− 0.16*	0.00^1^	− 0.10*	0.00^1^	− 0.12*	0.00^1^	− 0.13*	0.00^1^	− 0.16*	0.00^1^	− 0.04	0.12	− 0.13*	0.00^1^	− 0.15*	0.00^1^	− 0.18*	0.00^1^
Grade 9																		
SES	− 0.07	0.10	0.03	0.50	− 0.04	0.32	0.05	0.19	0.02	0.63	0.04	0.29	− 0.09	0.04	0.06	0.14	0.00	0.90
achievement	0.03	0.55	0.01	0.76	0.01	0.83	0.05	0.28	0.07	0.10	− 0.04	0.36	0.04	0.31	− 0.13*	0.00^1^	− 0.08*	0.04^1^

*Note*. *β* = standardized coefficients.

*p < .05;

^1^Significant at 0.05 with Type I error controlled (Benjamini–Hochberg correction).

### Teacher’s Perceived Challenges

Teachers were asked about the frequency of various challenges they encountered while planning students’ learning activities during the pandemic (Table 4). Some challenges were more serious than the others; repeated measure MANOVA, *F*(4, 384) = 26.44, *p* < .001. Generally, most teachers (61 − 79%) never experienced problems with a lack of support from the principal, middle management, school-based policy support measures, and technical staff. The most common challenge was the lack of computer skills. Even for this greatest challenge, only 10.5% and 6.6% of G.3 and G.9 teachers experienced such difficulty ‘many or every time’ they planned students’ learning activities.

**Table 4:** Distribution (%) Teacher’s Perceived Challenges During the Pandemic

Challenge: lack of	G.3					G.9				
	never	a few times	sometimes	many times	almost every time	never	a few times	sometimes	many times	almost every time
Principal support	74.6	16.7	4.5	2.3	1.9	79.2	12.8	4.0	2.8	1.2
Mid management support	69.8	19.3	5.7	3.2	2.0	71.0	16.4	7.4	4.6	0.6
School policy support	62.2	23.3	8.7	3.5	2.4	61.1	25.3	8.1	2.8	2.7
Computer skills	41.3	37.4	10.7	6.5	4.0	47.1	38.3	8.1	5.0	1.6
Skilled technical staff	61.0	26.4	8.1	2.4	2.1	61.0	26.4	7.0	3.9	1.6

*Note*. Items on 5-point scale (1 = never, 5 = almost every time)

### Students’ affect before and during the pandemic

We examined the changes in students’ four affects and whether they were related to their socioeconomic status and academic achievement using multilevel regression models (Table 5), with time (before, during COVID), SES, achievement, and their interactions as predictors. In G.9, high achievers were more lively but also more stressed (*β* = 0.20, 0.25 respectively, both *p* < .01), whereas in G.3, low achievers were more lively (*β* = − 0.14, *p* < .01).

**Table 5:** Multilevel Regression Predicting Students’ Affects with Time (before, during pandemic), SES, Achievement, Time × SES, Time × Achievement

	Happy			Lively			Worried			Stressed	
	*β*	*p*		*β*	*p*		*β*	*p*		*β*	*p*
**Grade 3**											
Time	− 0.01	0.29		0.07*	0.00		0.00	0.93		− 0.02	0.14
SES	− 0.01	0.77		0.05	0.10		0.02	0.44		− 0.03	0.36
Achievement	0.17*	0.00		− 0.14*	0.00		0.01	0.77		0.05	0.08
Time × SES	0.03*	0.03		− 0.04	0.05		0.01	0.39		− 0.02	0.31
Time × Achievement	− 0.02	0.26		− 0.01	0.62		− 0.01	0.31		− 0.03	0.03
**Grade 9**											
Time	− 0.08*	0.00		0.09*	0.00		− 0.14*	0.00		0.01	0.43
SES	− 0.08	0.04		0.04	0.32		− 0.02	0.54		− 0.07	0.10
Achievement	0.06	0.12		0.20*	0.00		− 0.01	0.88		0.25*	0.00
Time × SES	0.00	0.96		0.00	0.94		0.01	0.74		0.03	0.17
Time × Achievement	0.00	0.98		− 0.02	0.31		− 0.02	0.30		− 0.04	0.04

*Note*. *β* = standardized coefficients.

*p < .05; effects not significant after controlling for Type I error (Benjamini–Hochberg correction) are not marked as statistically significant.

Contradictory to the general expectation that COVID might have negative impacts on life, comparisons of the affects before and during COVID showed that students generally did not become less happy, more worried, or more stressed. Both G.3 and G.9 students actually felt more lively during COVID; *β* = 0.07, 0.09 respectively, both *p* < .01. Most importantly, low SES students’ affects were not affected more during the pandemic.

### Expected changes in academic achievement

Participants reported their expected change in students’ academic achievement upon returning to school (Tables 1; 1 worse than usual, 5 about the same, 9 better than usual). While all people might feel a drop in COVID, G.3 students felt their achievement was similar (5.39, 5.39, 5.87, all around 5). One-way ANOVA on each academic subject across the four groups of participants followed by posthoc comparisons showed (i) students were most optimistic (least drop), (ii) teachers were most pessimistic (largest drop), (iii) greater differences among the four groups in G.3 than in G.9, (iii) while G.3 students and parents felt a greater drop in Chinese and English than Maths, the other groups felt a slightly greater drop in Maths and English than in Chinese.

Multilevel regression analyses were conducted to examine whether low/high SES students and low/high achievers perceived bigger academic achievement changes (Table 6). Other than for G.9 Chinese and Maths (*beta* = − 0.03, 0.07, both *n.s.*), all G.3 and G.9 students with better academic achievement would perceive a smaller drop during COVID (G.3, 0.13, 0.20, 0.19 for Chinese, English, Math, all *p* < .01; G.9, 0.12 for English, *p* < .01). Importantly, low/high SES students did not perceive themselves to have a bigger drop, suggesting no evidence of worsening the social divide during the pandemic (G.3, − 0.02, 0.05, 0.00; G.9, 0.04, 0.03, 0.05 for Chinese, English and Mathematics respectively, all *n.s*.).

**Table 6:** Multilevel Regression Models on Students’ Expected Changes in Academic Achievement

	Expected Chinese Ach		Expected English Ach		Expected Maths Ach	
	*β*	*p*	*β*	*p*	*β*	*p*
**Grade 3**						
Chinese language						
SES	− 0.02	0.42				
Ach	0.13	0.00				
English language						
SES			0.05	0.07		
Ach			0.20	0.00		
Mathematics						
SES					0.00	0.91
Ach					0.19	0.00
**Grade 9**						
Chinese language						
SES	0.04	0.33				
Ach	− 0.03	0.33				
English language						
SES			0.03	0.50		
Ach			0.12	0.00		
Mathematics						
SES					0.05	0.17
Ach					0.07	0.06

*Note*. Ach = Achievement; *β* = standardized coefficients

### Life satisfaction during the pandemic

To understand the effect of the pandemic on students’ life satisfaction, we compared students’ life satisfaction during the pandemic against responses from another representative batch of students collected a year before the pandemic with t-tests (Table 7). Using effect sizes of 0.2, 0.5, 0.8 as small, medium, and large effects (Cohen, 1988), students had a medium drop in ‘satisfaction in school learning’ (*d* = 0.37, 0.24 for G.3, G.9), a large drop in ‘satisfaction in friendship’ for G.3 students (0.51), and a medium increase in ‘satisfaction in health’ for G.9 students (0.34). Otherwise, COVID did not lead to any substantial drop (or improvement) in life satisfaction.

**Table 7:** *Life Satisfaction of Two Batches of Students Before and During the Pandemic*

	Before Pandemic (N = 10,064)		During Pandemic (N = 2,019)				
	*M*	*SD*	*M*	*SD*	*t*	Δ Score	*d*
**Grade 3**							
Health	3.35	0.61	3.43	0.71	-4.85	-0.08*	− 0.12
Learning at school	3.41	0.63	3.16	0.73	14.59	0.25*	0.37
Friends	3.51	0.66	3.10	0.92	19.07	0.41*	0.51
Things you have	3.46	0.67	3.35	0.82	5.73	0.11*	0.15
Time	3.17	0.71	3.04	0.79	6.55	0.13*	0.17
Relation w/parents	3.54	0.64	3.48	0.74	3.12	0.06*	0.09
Relation w/teachers	3.31	0.66	3.22	0.75	4.91	0.09*	0.13
	(N = 4445)		(N = 1653)				
	*M*	*SD*	*M*	*SD*	*t*	Δ Score	*d*
**Grade 9**							
Health	3.03	0.61	3.25	0.69	-11.75	-0.22*	− 0.34
Learn at school	2.99	0.56	2.84	0.70	7.93	0.15*	0.24
Friends	3.20	0.60	3.17	0.73	1.67	0.03	0.04
Things you have	3.15	0.61	3.19	0.70	-1.95	-0.04	− 0.06
Time	2.79	0.69	2.93	0.75	-6.56	-0.14*	− 0.19
Relation w/parents	3.18	0.63	3.29	0.66	-6.17	-0.11*	− 0.17
Relation w/teachers	3.09	0.54	3.06	0.61	1.31	0.03	0.05

*Note*. *d* = effect size

*p < .05

We also examined whether life satisfaction of SES-disadvantaged students and low academic achievers declined more than their peers during the pandemic using multilevel regression models (with Benjamini–Hochberg correction for Type I error). Low (or high) SES students were not more (or less) dissatisfied with various aspects of life during COVID.

## Discussion

The pandemic forced all educational systems to adopt a remote teaching mode without much preparation. It also tested out how much students could learn without daily monitoring and assistance from their teachers. The IT provision at home, IT support for teachers, students’ competence in e-learning, students’ self-monitoring/self-discipline to learn with less supervision, and teachers’ readiness to adopt an appropriate pedagogy, among others, were crucial in ensuring the success of emergency remote teaching. The present study assessed and compared students, parents, teachers, and principals on their perceived students’ challenges, affects, life satisfaction, and expected academic performance changes during COVID. The possible deteriorations of the social and digital divide were also examined.

### Life as Usual for students in Hong Kong

Generally, teachers, principals, and parents were quite worried that students could not survive without the help of adults (lack of concentration and teachers’ explanation). Teachers and principals were also very concerned about the learning situation (lack of computer/stable internet, quiet place to study). In contrast, students, particularly the younger ones, did not find them challenging. They perceived themselves to be livelier during COVID. G.3 students also felt their academic achievement to be unaffected by COVID. This was in big contrast to the much greater worry among the teachers.

One of the public greatest concerns was whether the social and digital divide had become more serious. Results showed that the ‘lack of computers/stable internet’ was not a serious concern by all parties, particularly among the students and parents. Results also showed that the socially disadvantaged students generally did not have more severe challenges; they were not more negative regarding affects, life satisfaction, or perceived academic achievement.

### Conducive learning environment

Results of the present study using parallel sets of questionnaire items on various stakeholders suggested that Hong Kong students did not perceived the COVID impacts on their learning and affects as negative as most of their teachers, principals or parents might have expected. The COVID impacts reported here were also much less negative than the majority of literature we have reviewerd. There were many possible reasons for the seemingly low negative impact on Hong Kong students. First, Hong Kong is a metropolitan city with high availability of computers (laptops or other devices) and stable internet ready to support learning ‘as usual’ within a short time (Ng et al., 2020). The teacher-to-student ratio (Hong Kong 1:13.5, Hau 2017) was on par with all major developed economies (OECD average 1:13.1, OECD, 2016), and all new primary and secondary school teachers had to be degree holders (Hong Kong Education Bureau, 2021).

One primary public concern was whether students had enough computers and internet support during the pandemic when most teaching had been shifted to the internet. Relatively Hong Kong is an economically developed city with an outstanding education equity system (OECD, 2019); this possibly explains why the lack of computers and stable internet was not seen by all people (students, parents, teachers, principals) as the most critical challenge during the pandemic.

On COVID infection, Hong Kong did not have a lot of identified and death cases relative to many other economies. In the Hong Kong population of around 7.5 million, there were only around 3,400 locally identified cases, with 31 fatal cases up to the first half-year (to August 1, 2020). So other than social distancing throughout the whole period, some work-from-home, and the closing of restaurants in short periods, the city had been relatively unaffected by the pandemic before and during the data collection.

Actually, in a society where education and academic achievement have been very strongly emphasized (Hau & Ho, 2010), a much slower teaching pace, without weekly quizzes or tests and examinations in remote teaching, can provide a more relaxing life to all students. While parents and teachers worry about academic progress, students may not feel the same as long as there are no high-stakes examinations to monitor their learning.

The social or digital divide did not seem to have widened in Hong Kong. First, Hong Kong has high education equality and is second-best in large-scale international comparisons (e.g., OECD, 2019). Second, computers/laptops and stable internet were available in most families, with schools ready to lend laptops and provide free Wi-Fi to students in need.

### Lessons from Emergency Remote Teaching

Three insights emerged in OECD EPO analyses of many educational systems during the pandemic. Specifically, they were ‘learning does not need to be constricted within the four walls of an educational institution,’ ‘education systems are not too heavy to move and … education actors can reach agreements that can make significant change happen in education,’ and ‘only resilient education systems… will be able to fulfil the fundamental human right to education, whatever the circumstances, and foster the level of human capital required for successful economies and societies’(OECD., 2020, p. 11).

Regarding learning pedagogy and technology, Andreas Schleicher (in OECD, 2020) pointed out that COVID has accelerated our thinking on how technology can be used in education. The possible impacts are (i) the role of technology in future education, (ii) the multi-dimensional function of formal education, (iii) learning (schooling) is an activity rather than a place, and (iv) the changing purpose of assessment when standardized assessment became difficult. Understandably the closure of schools and social distancing forced students out of their schools, the primary place where learning takes place.

In promoting the use of technology in teaching and learning, an immediate concern is whether we would widen the digital divide. Thus, there have been calls for more help for disadvantaged students to avoid exacerbating the digital divide disparities during the pandemic (Bozkurt et al., 2020; Dodd et al., 2021). Common solutions are to launch free digital device rental services (Ministry of Education Republic of Korea, 2020) and provide high-speed internet (Japan Ministry of Education, Culture, Sports, Science and Technology, 2020).

We learn from the pandemic that providing the necessary IT support and a quick switch to an appropriate and efficient emergency remote teaching is essential. Perhaps an even more important educational goal is nurturing students who can be resistant to various challenging learning situations (OECD, 2020).

## Data Availability

The data that support the findings of this study are available from the Hong Kong Education Bureau, but restrictions apply to the availability of these data, which were used under license for the current study, and so are not publicly available. Data are, however, available from the authors upon reasonable request and the permission of the Hong Kong Education Bureau.
